# RNA interference targeting virion core protein ORF095 inhibits Goatpox virus replication in Vero cells

**DOI:** 10.1186/1743-422X-9-48

**Published:** 2012-02-17

**Authors:** Zhixun Zhao, Guohua Wu, Xueliang Zhu, Xinmin Yan, Yongxi Dou, Jian Li, Haixia Zhu, Qiang Zhang, Xuepeng Cai

**Affiliations:** 1 Key Laboratory of Animal virology of the Ministry of Agriculture, State Key Laboratory of Veterinary Etiological Biology, Lanzhou Veterinary Research Institute, Chinese Academy of Agriculture Sciences, No. 1 Xujiaping, Lanzhou, Gansu 730046, PR China; 2 Lanzhou Veterinary Research Institute, Chinese Academy of Agriculture Sciences, No. 1 Xujiaping, Lanzhou, Gansu 730046, PR China

**Keywords:** RNAi, shRNA, ORF095, Goatpox virus

## Abstract

**Background:**

Goatpox is an economically important disease in goat and sheep-producing areas of the world. Many vaccine strategies developed to control the disease are not yet completely successful. Hairpin expression vectors have been used to induce gene silencing in a large number of studies on viruses. However, none of these studies has been attempted to study GTPV. In the interest of exploiting improved methods to control goat pox, it is participated that RNAi may provide effective protection against GTPV. In this study we show the suppression of Goatpox virus (GTPV) replication via knockdown of virion core protein using RNA interference.

**Results:**

Four short interfering RNA (siRNA) sequences (siRNA-61, siRNA-70, siRNA-165 and siRNA-296) against a region of GTPV ORF095 were selected. Sense and antisense siRNA-encoding sequences separated by a hairpin loop sequence were designed as short hairpin RNA (shRNA) expression cassettes under the control of a human U6 promoter. ORF095 amplicon was generated using PCR, and then cloned into pEGFP-N1 vector, named as p095/EGFP. p095/EGFP and each of the siRNA expression cassettes (p61, p70, p165 and p296) were co-transfected into BHK-21 cells. Fluorescence detection, flow cytometric analysis, retro transcription PCR (RT-PCR) and real time PCR were used to check the efficiency of RNAi. The results showed that the ORF095-specific siRNA-70 effectively down-regulated the expression of ORF095. When Vero cells were transfected with shRNA expression vectors (p61/GFP, p70/GFP, p165/GFP and p296/GFP) and then infected with GTPV, GTPV-ORF095-70 was found to be the most effective inhibition site in decreasing cytopathic effect (CPE) induced by GTPV. The results presented here indicated that DNA-based siRNA could effectively inhibit the replication of GTPV (approximately 463. 5-fold reduction of viral titers) on Vero cells.

**Conclusions:**

This study demonstrates that vector-based shRNA methodology can effectively inhibit GTPV replication on Vero cells. Simultaneously, this work represents a strategy for controlling goatpox, potentially facilitating new experimental approaches in the analysis of both viral and cellular gene functions during of GTPV infection.

## Background

GTPV is a member of the Genus Capripoxvirus of the family Poxviridae [1], which also includes the Sheeppox virus (SPPV) and the Lumpy Skin Disease Virus (LSDV) of cattle. Both sheeppox and goatpox are endemic in Africa, the Middle East and many countries in Asia, and the diseases caused by these viruses have a significant economic impact on the livestock industry in Africa and Asia [2]. GTPV genome is approximately 150 kbp double-stranded DNA, which composes at least 147 putative genes, including conserved replicative and structural genes and genes likely involved in virulence and host range [3]. ORF095 encodes the virion protein which constitutes a great part of the total protein content of the virion and is essential during the assembly and disassembly of virion. It is similar to myxoma virus (MYXV) M093L (accession no.AF170726) [4,5] and vaccinia virus (VACV) A4L (accession no.M35027) that encodes a 39 kDa acidic protein, a part of the viral core, and is synthesized at late stages after infection [6,7].

RNA-mediated interference (RNAi) is a conserved gene-silencing mechanism, where by the double-stranded RNA matching is used as a signal to trigger the sequence-specific degradation of homologous mRNA [8]. RNAi can be triggered by chemically synthesised and enzymatically produced 21-25 nt long RNA duplexes in mammalian cells [9,10]. Since the effect of short interfering RNAs (siRNAs) is generally temporal in transfected animal cells, small RNA expression vectors have been developed to induce long-lasting RNA silencing in mammalian cells [11-14]. RNAi represents a new antiviral method and is being increasingly used to inhibit the replication of viral pathogens [15] such as foot-and-mouth disease [16,17], porcine reproductive and respiratory syndrome virus [18], Newcastle disease virus [19], classical swine fever virus [20] and Monkeypox virus [21]. Hairpin expression vectors have been used to induce gene silencing in a large number of studies on viruses [11,22-26].

This study provides not only an experimental basis for the development of a new anti-GTPV strategy, but also for a new approach to the study of GTPV infection and replication.

## Materials and methods

### Viruses and cells

Goatpox virus strain A/Goat/Qinghai/AV40/2006(a cell-adapted strain) was used in this study and maintained in African green monkey kidney cells (Vero). Baby Hamster Kidney cells (BHK-21) and the GTPV permissive cell line Vero (Lanzhou Veterinary Research Institute, Chin) were cultured in Dulbecco's modified Eagle's medium (DMEM; Sigma) supplemented with 10% heat-inactivated fetal bovine serum (FBS; Hangzhou, China), 100 U/ml penicillin and 100 μg/ml streptomycin (Sigma). Cultures were incubated at 37°C with 5% CO2.

### Construction of plasmids

**Figure 1:**
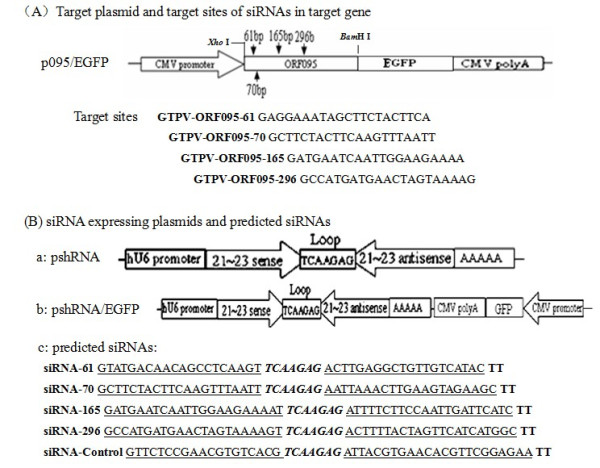
**Schematic representations of target construct, siRNA-expressing plasmids, and predicted siRNAs**. (A)Position of target siRNAs: GTPV-ORF095-61, GTPV-ORF095-70, GTPV-ORF095-165 and GTPV-ORF095-296. (B) siRNA expressing plasmids and predicted siRNAs. An inverted repeat is inserted at the 3'-end of the human U6 promoter. The forward sequence of the repeat is 21 or 63 nt long, corresponding to the region of interest of the ORF095 gene. The forward and reverse motifs are separated by a 7-nt spacer, 5'-TCAAGAG-3'. The transcriptional termination signal of five Ts is added at the 3'-end of the inverted repeat. The synthesized RNA is predicted to fold back to form a hairpin dsRNA, which would be finally processed into the putative siRNAs.

The cDNA cassettes corresponding to the conserved gene of the GTPV genome was cloned into the pEGFP-N1 vector (Figure 1A). Directed cloning PCR was used to amplify the ORF095 gene, using the following primers (sense: 5'-GTCCTCGAGATGGACTTCATGAAAAAATATACTAA-3' and antisense: 5'-GCGGATCCTTGCTGTTATTATCATCTAGTTTG-3') used for amplification contained the target sequences for *Xho*I (CTCGAG) and *Bam*HI (GGATCC) incorporated at the 5' of the viral complementary sequence. Forward primer contained an ATG sequence, before the sequence that codified for the protein, as a start initiation codon of protein translation. The reverse primer uncontained a TTA sequence, which was used in the characterization of ORF095 protein and EGFP co-expression. PCR products were digested with *Xho*I and *Bam*HI, and cloned into the pEGFP-N1 expression vector (Invitrogen, Inc., Shanghai, China). The final construct p095/EGFP (Figure 1A) was analyzed by restriction digestion and sequenced. Plasmids used for transfection were purified with the Wizard Purefection TM Plasmid DNA Purification System (Promega, USA) and quantified by Biophotometer (Eppendorf, Germany).

### Target sequence selection and vector construction

As the AAGN18_UU _sequence (N, any nucleotide) has been found to be preferred for siRNA-mediated gene silencing under the control of the PolU6 promoter [27], we searched for this sequence in the ORF of ORF095 gene. Four fragments (ORF095-61, ORF095-70, ORF095-165 and ORF095-296) in the coding region of ORF095 gene were selected according to the web-based criteria (http://www.ambion.com). These selected sequences were then submitted to a BLAST searching against human genome sequence to check whether or not these potential targets have homologues in the human genome was not targeted. To construct hairpin siRNA expression cassette, the following DNA oligonucleotides were synthesized: GTPV-ORF095-61, GTPV- ORF095-70, GTPV-ORF095-165 and GTPV-ORF095-296 (Figure 1A). The 21 nt target sequences served as a basis for the design of the two complementary 51-53mer siRNA template oligonucleotides that were synthesized, annealed, and inserted into *Bam*HI and *Bbs*I sites of the siRNA expression vectors pGPU6/Neo and pGPU6/GFP/Neo (GenePharma Co., Ltd, Shanghai, China), respectively. The recombinant plasmids were designated as p61, p61/GFP, p70, p70/GFP, p165, p165/GFP, p296 and p296/GFP. The pC and pC/GFP negative controls (GenePharma Co., Ltd, Shanghai, China) were negative control plasmid, which encode hairpin siRNA that does not have homologues in mice, human and Carpripoxvirus genome databases.

### Transfection of the siRNA expression cassettes into BHK-21 cells

BHK-21 cells were seeded in six-well plates and cultured at 37°C and 5% CO2 overnight. When the cells showed 70-80% confluence, 2.5 μg of p61, p70, p165 or p296 each were cotransfected with an equal amount of p095/EGFP using FuGENE HD Transfection Reagent (Roche, Germany) according to the Manufacturer's recommendations, respectively. Simultaneously, 2.5 μg of p095/EGFP were cotransfected with 2.5 μg of pC. Non-transfected BHK-21 cells were also used as a control.

### Analysis of EGFP expression in BHK-21 cells and flow cytometry assay

After an additional 24 h of incubation, cells were observed for the expression of green fluorescent protein in the transfected cells was monitoring fluorescent microscope (Olympus, Japan).

Cells were further subjected to fluorescence-activated cell sorting (FACS). At 48 h posttransfection, the transfected cells and the controls were washed gently in phosphate-buffered saline (PBS), trypsinized and resuspended in PBS. EGFP positive cells and EGFP expression signal were evaluated by the FACS Calibur Flow Cytometry System (Becton Dickinson, USA).

### Reverse transcription (RT)-PCR

To confirm the efficacy of RNAi, RT-PCR was used to amplify the target gene in the transfected cells. Total RNA was extracted from BHK-21 culture with Trizol reagent (TaKaRa, Dalian), and incubated for 1 h at 37°C with Dnase RQ1 (TaKaRa, Dalian). To detect ORF095 mRNA expression in BHK-21 cells, RT-PCR was conducted using 1.8 μg of RNA extracts with Superscript one-step RT-PCR system (Gibco, BRL). Retrotranscription β-actin as a control was also amplified using the Primers 5'-CACCCGCGAGTACAACCTTC-3' (sense) and 5'-CCCATACCCACCATCACACC-3' (antisense). PCR was run for 30 cycles with 95°C for 30 s, 56°C for 45 s and 72°C for 45 s. To verify primer specificity, a melting curve was analyzed, and RT-PCR products were further cloned into pMD18-T for sequencing.

### Real-time PCR analysis

In order to use full-length ORF095 gene as a quantitative RT-PCR standard, Selected primer sequences were CTGTCTACATGATTAACCCACTCGTTCTTC (ORF095 FP primer), and GAAGTCGACTAC CCCTCTCCCTATCAGGGTCATC (ORF095 RP primer). One additional primer was synthesized for quantification of the ORF095 in real-time PCR: 5-FAM-CCTTGCTCGCGAATTTCTCACCGATAMRA- 3 (TaqMan probe). The target region of real time RT-PCR was 263-389 bp of ORF095.

For quantitative analysis of the ORF095 gene, 100 ng total RNA from p095/EGFP-transfected cells was mixed with 1 μL ORF095 primer, heated to 65°C for 5 min and chilled on ice for 2 min. To this primer template mix was added 5× buffer (4 μL), 10 mmol/μL dNTP (1 μL), RNasin (1 μL), AMV reverse transcriptase (1 μL, Promega, USA) and ddH_2_O to a total volume of 20 μL. The reaction mixture was incubated at 42°C for 45 min, followed by inactivation of reverse transcriptase at 75°C for 15 min. Real-time PCR was performed with the ABI PRISM 7000 Sequence Detection System using 2 μL transcriptase products as template under the conditions of 95°C for 15 min, followed by 50 cycles of denaturation at 95°C for 30 s, annealing, and extension at 60°C for 30 s. The quantitative standard curve for determination of ORF095 copy number was created by real-time PCR of standard plasmid p095/EGFP serial 10-fold dilutions of a stock containing 10^8 ^copies/μL. The specificity of the real-time PCR was confirmed by sequencing of the product.

### Transfection and GTPV infection Vero cells

Four siRNAs targeting ORF095 were designed to inhibit GTPV ORF095 gene expression in BHK-21 cells. We used a modified CMV promoter, a typical RNA polIII promoter, to drive the transcription of the siRNAs. To monitor the effects of the siRNAs, eukaryotic expression plasmid p095/EGFP was constructed, in which the ORF095 gene were fused to the 5'-end of the EGFP coding sequence, and cotransfected with their specific siRNA expression plasmids. So the inhibitory effects of the ORF095-specific siRNAs on the ORF095 expression could be indirectly evaluated by the expression of EGFP in the transfected cells.

To test whether the expressed siRNAs inhibited GTPV production, we first assessed the growing capacity of GTPV in Vero cells expressing siRNAs. Vero cells were seeded in six-well plates and cultured at 37°C and 5% CO_2 _overnight. When the cell reached 70-80% confluence, 2.5 μg of p61/GFP, p70/GFP, p165/GFP or p296/GFP each were cotransfected with an equal amount of pC/GFP as described above, respectively. The nonspecific vector pC/GFP and non-transfected Vero cells were also used as a control. 24 h posttransfection, the cells were infected with GTPV at a multiplicity of infection (moi) of about 0.01. Briefly, after removing the culture medium, GTPV (200 μl) in infection medium (2.5 μg/ml trypsin), respectively, were added to each well. The cultures were then incubated at 37°C, 5% CO_2 _for 4 h, at which point the culture medium was replaced with fresh DMEM containing 2% fetal bovine serum.

### Virus titration

To determine transfection efficiency, we monitored GFP fluorescence intensity of transfected cells using fluorescent microscope analysis. Culture supernatants were collected for virus titration. Six days post infection, supernatants was harvested from the infected cultures and virus titer (TCID50) was determined three times on Vero cells.

Virus infectivity was determined by serial dilutions of the samples in 96-well plates and the virus titer was calculated as a TCID50 by the Reed-Muench method [28]. A viral suspension titrated at 10^-1 ^to 10^-8 ^TCID50 per 0.1 ml was used for viral challenge. Vero cells (about 80% confluent) grown in 96-well plates were transiently transfected with 0.1 μg p61/GFP, p70/GFP, p165/GFP and p296/GFP, respectively, per well. After 6 h of transfected, the transfection medium was removed and the cells were washed twice with DMEM medium. The transfected cells were then infected with 100 TCID50 of virus per 0.1 ml per well. After additional 1 h incubation, the inoculum was removed and the cells were washed twice with DMEM medium. The cells were then maintained in DMEM medium supplemented with 10% fetal bovine serum for 6 days. For detecting the therapeutic potential of siRNA, in another parallel experiment, transfection was performed 1 h post-infection with the virus. GTPV replication in Vero cells was evaluated by virus titer (TCID50).

## Results

### Transient cellular transfection and analysis of the targeted gene and EGFP expression in BHK-21 cells

**Figure 2:**
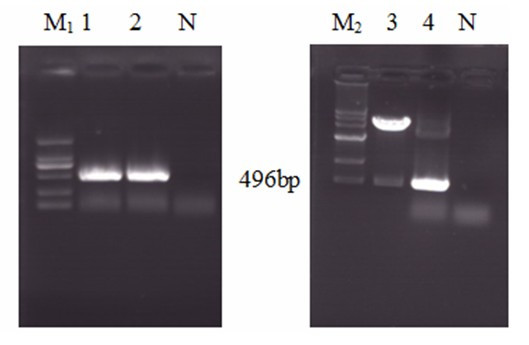
**Amplification of ORF095 gene from the GTPV genome by PCR and identification of recombinant plasmid p095/EGFP by restriction and PCR**. M_1 _DNA marker DL 2000; 1&2 PCR products; M_2 _DNA marker 500-12000; 3 map of restriction by *Bam*HI and *Xho*I; 4 PCR product; N negative control.

**Figure 3:**
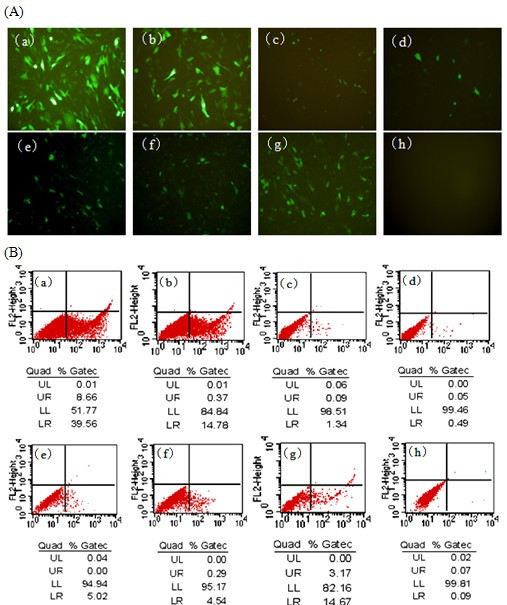
**Transient expression of siRNAs conferred the sequence-specific inhibition of expression of GTPV ORF95 in BKH-21 cells**. (A) Fluorescence detection of cotransfection of p095/EGFP with their corresponding siRNA expression plasmids 24 h posttransfection. (B) Flow cytometric analysis of cotransfection of p095/EGFP with their corresponding siRNA expression plasmids 48 h posttransfection. EGFP expression level in cells cotransfected with (a) pEGFP-N1 vector; (b) p095/EGFP; (c) p095/EGFP and p61; (d) p095/EGFP and p70; (e) p095/EGFP and p165; (f) p095/EGFP and p296; (g) p095/EGFP and pControl; (h) BHK-21 cell control. LR-Value means the rate of EGFP positive cells.

Different siRNAs suppressed the expression of fusion green fluorescent protein in BHK-21 cells is different. The siRNAs targeting to the conserved region of GTPV genome were generated in vitro by human recombinant Dicer enzyme, as described in Figure 1B. To identify an effective inhibitory effect of siRNAs, the cDNA cassettes of these regions were inserted into the 5' end of enhanced green fluorescent protein (EGFP) gene to construct reporter plasmids (Figure 2). The reporter plasmids were used to cotransfect BHK-21 cells with either the homologous siRNAs or the heterologous siRNAs. The results showed that the number of EGFP-expressing cell was markedly reduced in the sample transfected with homologous siRNAs than sample transfected with heterologous siRNAs or non-transfected (Figure 3A). FACS demonstrated that the levels of inhibition mediated by the siRNAs were similar among the different experiment groups and significantly higher than the control group (cotransfection with heterologous siRNAs or without siRNAs).

The inhibitory effects of the siRNAs on expression of EGFP were quantitatively validated by flow cytometry 48 h posttransfection. The extent of EGFP down regulation was assessed by the mean fluorescence of the positive cells (LR-values) and the rate of EGFP positive cells (Figure 3B). Compared with the pC, the LR-values of the EGFP positive cells were reduced in the cells transfected with ORF095 siRNA-specific expression plasmids p61, p70, p165 and p296, and were reduced by 90.9%, 96.7%, 66.0%and 69.32%, respectively.

**Figure 4:**
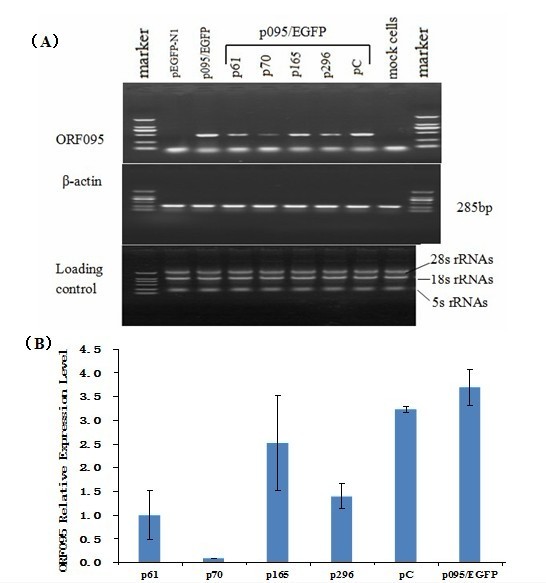
**Analysis effect of RNAi on ORF095 by RT-PCR**. (A) Cells were harvested, and transcripts were analyzed by RT-PCR amplification. The 28S, 18S, and 5.8S rRNAs were visualized under UV light for equal loading control. (B) Specific siRNA inhibits the accumulation of ORF095 message. BHK-21 cells were transfected with pEGFP-N1 and then transfected with variant shRNA-expressing vectors. Forty hours post-transfection, total RNA was extracted and subjected to fluorescence quantitative PCR analysis. pC transfected cells and mock-transfected cells were used as controls. The mRNA of beta actin served as an internal reference.

To further demonstrate the levels of inhibition, cells were collected 48 h post-transfection and RT-PCR analysis was performed. The level of target RNA, as determined by RT-PCR, was also significantly reduced in cells transfected with homologous siRNAs (Figure 4A). To measure the level of gene suppression accurately, QPCR primers and Taqman probes directing to ORF095 were designed. We also designed probes and primers directed toβ-actin sequence (serve as internal reference). When normalized for loading differences using the β-actin mRNA, the ORF095 message in the cells transfected with p61, p70, p165 and p296 were reduced by 69%, 97%, 22%and 57% (ORF095 message copies ratios of cells transfected with shRNA expression vectors/cells transfected with empty vector)(Figure 4B). There was no significant inhibition in cells transfected with the empty vector pEGFP-N1 and nonspecific shRNA expression vector pC. mRNA levels of ORF095 (average ORF095 mRNA levels in cells treated with p61, p70, p165, p296, pC and empty vector were 0.416, 0.036, 1.046, 0.580, 1.345, respectively) or β-actin suggested that the reductions in ORF095 message did not result from poor transfection, nonspecific inhibition or toxicity, because the average mRNA levels of β-actin for experimental cells were not significantly reduced compared to the control cells. In addition, the suppressive effect was found to be gene-specific, because the inhibitory effect of empty vector and nonspecific shRNA expression vector pC were negligible. These results suggest that the siRNA generated by in vitro transcription effectively and specifically inhibit the expression of GTPV ORF095 conserved regions in BHK-21 cells.

### Interference of GTPV replication by shRNA expression vector

**Figure 5:**
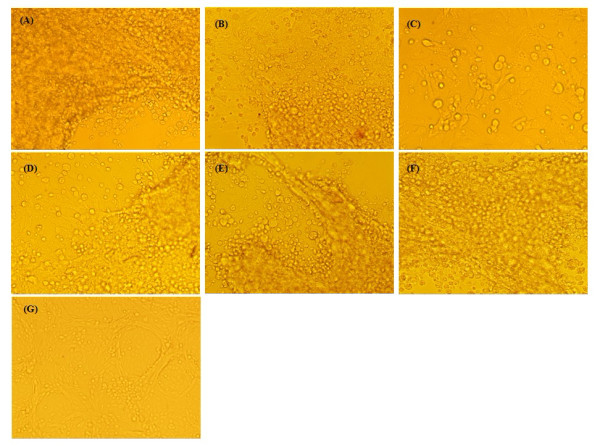
**Cytopathic effect (CPE) analysis of GTPV on Vero cells transfected with shRNA-expressing vectors (2.5 μg each)**. (A):Cells just were infected GTPV; (B)-(G):p61/GFP, p70/GFP, p165/GFP, p296/GFP, pC/GFP and untreated cells, respectively. Except(G), all cells were infected with GTPV(A/Goat/Qinghai/SV40/2006) isolate at a multiplicity of infection (MOI) of 0.01. Pictures were taken at 6 days post-infection with an Olympus digital camera (Olympus, Japan) at a magnification of × 40 with an exposure time of 1/8 s.

To investigate whether or not knockout of ORF095 relieves cytopathic effect (CPE) induced by GTPV, Vero cells were transfected by plasmids expressing ORF095 protein-targeted shRNAs (p61/GFP, p70/GFP, p165/GFP and p296/GFP), respectively. Nonspecific shRNA expression vector pC/GFP was transfected in parallel. 4 h post-transfection, the cultures were infected with GTPV and checked daily. Six days later, we found that cells pre-transfected with p70/GFP exhibited less CPE, whereas other shRNA-treated cells and the empty vector control demonstrated the same typical GTPV-induced CPE as cells infected only with virus, as shown in Figure 5.

**Figure 6:**
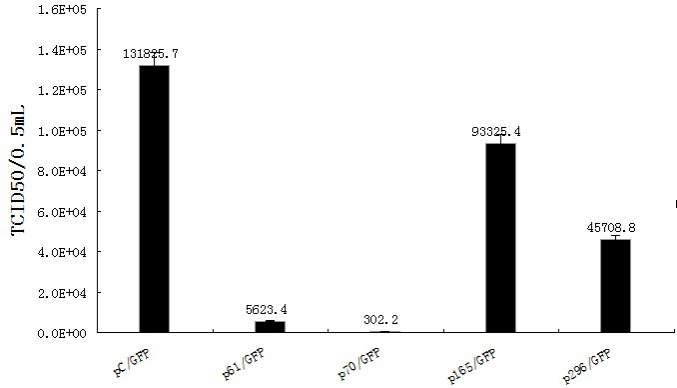
**GTPV-ORF095 gene RNA-specific siRNAs can inhibit the production of progeny virus**. Vero cells transiently transfected with empty vector or plasmid carry siRNA-61, 70, 165 or 296 were infected with GTPV at an MOI of 0.01. Viruses were harvested and titers were determined as described above. Mock-infected cells were used as a control. The values given are average of three independent experiments. Error bars indicate standard deviations.

The TCID50 assay was performed to examine the effect of siRNA on production of viable virus, and the results (Figure 6) showed that in control cells the titers reached 10^-5.12 ^TCID50/0.1 mL at 6 days post-infection. In contrast, the titers in the cells transfected with p61/GFP, p70/GFP, p165/GFP or p296/GFP, were 10^-3.75^, 10^-2.48^, 10^-4.73 ^and 10^-4.66^/0.1 mL 6 days post-infection respectively, corresponding to 23.4-, 463.5-, 1.4- and 2.9- fold reductions in viable virus production.

### Dose-dependent inhibitory effect of shRNA expression vector p70/GFP

**Figure 7:**
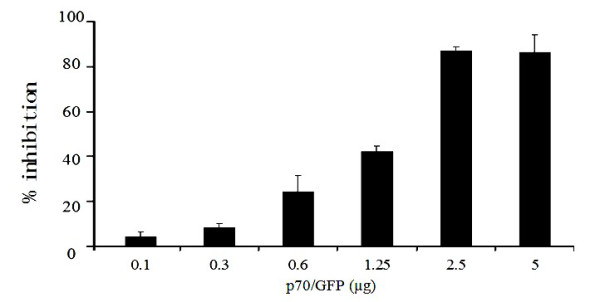
**Dose-dependent inhibitory effect of shRNA expression vector p70/GFP**. Vero cells were transfected with variant p70/GFP and then infected with GTPV at an MOI of 0.01. 48 h post-infection, total RNA was extracted and subjected to quantitative PCR analysis. Mock-transfected cells were used as controls. The mRNA of beta actin served as an internal reference. Black bars indicate normalized shRNA+ (cells transfected with p70/GFP at six different concentrations ranging from 0.1 to 5.0 μg) GTPV message copies ratios. The data shown represent average from three experiments with the standard deviations indicated by error bars.

To characterize the antiviral properties of most potent siRNA construct, namely p70/GFP, Vero cells were transfected with dilution of each construct to cover a range of 0.1-5.0 μg in six concentrations. Overnight transfected cells were infected with GTPV at an MOI of 0.01 and viral replication was examined at 48 h PI (Figure 7). p70/GFP showed average viral replication inhibition of 5.3% at the lowest tested concentration of 0.1 μg, and complete inhibition at concentrations of 2.5 μg and higher.

## Discussion

RNAi is a process of sequence-specific, post-transcriptional gene silencing that is initiated by double stranded RNA. Introduction of siRNA results in degradation of siRNA specific transcripts thus reducing the expression of their protein product. In plants, it is a natural antiviral defense mechanism [29]. In mammalian cells, however, dsRNAs longer than 30 nt activate an antiviral defense leading to the nonspecific degradation of RNA transcripts and a general shutdown of host-cell protein translation [30]. The successful use of siRNA in animal cells encouraged the development of siRNA expression vector [31] and numerous studies have demonstrated that DNA-based siRNA is a promising approach for antiviral therapy in mammals. RNAi represents a new antiviral method and is being increasingly used to inhibit the replication of viral pathogens [15], such as HIV-1 [32], hepatitis C [33], influenza [34], severe acute respiratory syndrome [35]and hepatitis E viruses [36]. This study has demonstrated the use of pGPU6/Neo or pGPU6/GFP/Neo vector-based RNAi against GTPV, a major pathogen of goats and sheep. Four different siRNA targeting viral gene ORF095, one key gene involved in GTPV replication, successfully reduced viral replication.

The results showed that the ORF095-specific siRNAs p70 could effectively down-regulate the expression of ORF095, while p61, p165 and p296 displayed weak activity. Additionally, expression of the housekeeping gene β-actin was also analyzed by RT-PCR and quantitative real-time PCR, and no significant difference in the expression of β-actin was observed between the siRNAs treatment groups and pC treatment groups.

Different siRNA sequences display widely different efficacies with regard to suppression of gene expression, requiring screening of multiple sequences [37]. In this research, we have selected four target sequences for RNA interference by the software applications, "siRNA Target Finder and Design Tool" available at http://www.ambion.com/. As the ORF095 gene is well conserved in GTPV and ORF095 protein is a virion core protein and assembly protein in GTPV, we selected ORF095 as a target gene. In order to generate shRNA expression cassettes quickly and accurately, we employed a PCR-based strategy to clone siRNA sequences. In this strategy, siRNA sequences were designed as a single primer sequence of which 19 bp complementary to the human U6 promoter were added. The resulting PCR products are shRNA expression cassettes including the human U6 promoter. shRNAs that have been generated from this expression system are efficiently processed by dicer into siRNAs. In addition, in this study, we selected pEGFP-N1 vector that contains an EGFP gene as report gene and can be transfected into mammalian cells using any standard transfection method.

Vero cells transfected with p61/GFP, p70/GFP, p165/GFP, p296/GFP and pC/GFP were examined for CPE by virus titration. All results demonstrated that siRNA-70 is the most effective one, and result showed that minimum concentration of the construct p70/GFP is 2.5 μg required to induce maximum inhibition.

Blasting ORF095 sequence in GenBank revealed that there were 8 Capripoxvirus isolates containing identical sequence corresponding to ORF095. In view of the sequences of the ORF095 genes of GTPV strains from the same genotype, they all share high homology (95-100%). Therefore, ORF095 gene is a good target to suppress GTPV replication by RNAi.

## Conclusion

In conclusion, this study demonstrates that vector-based shRNA methodology can effectively inhibit GTPV replication on Vero cells. Further study will be required to determine whether such treatment protect against GTPV infection in vivo. Still, this work represents a strategy for controlling goatpox, potentially facilitating new experimental approaches in the analysis of both viral and cellular gene functions during of GTPV infection.

## Abbreviations

GTPV: Goatpox virus; RNAi: RNA-mediated interference; siRNA: Short interference RNA; shRNA: Short hairpin RNA; GFP: Green fluorescent protein; EGFP: Enhanced green fluorescent protein; p095/EGFP: pEGFP-N1 expression ORF095 gene cassette; p61 p70, p165, p296 and pC were siRNA-61, 70, 165, 196 and control expression cassettes pGU6/Neo plasmids; p61/GFP p70/GFP: p165/GFP, p296/GFP and pC/GFP were siRNA-61, 70, 165, 196 and control expression cassettes pGU6/GFP/Neo plasmids; CPE: Cytopathic effect; PCR: Polymerase chain reaction; RT-PCR: Retro transcription PCR; SPPV: Sheeppox virus; LSDV: Lumpy Skin Disease Virus; MYXV: Myxoma virus; VACV: Vaccinia virus; BHK-21: Baby Hamster Kidney cells; Vero: African green monkey kidney cells; DMEM: Dulbecco's modified Eagle's medium; FBS: Fetal bovine serum; FACS: Fluorescence-activated cell sorting; PBS: Phosphate-buffered saline; MOI: Multiplicity of infection; PI: Post-infection.

## Competing interests

The authors declare that they have no competing interests.

## Authors' contributions

QZ and XC designed research; ZZ performed research and wrote the paper; GW helped to construct partial plasmids and analyzed data; XZ contributed new reagents/analytic tools; XY provided partial plasmids. HZ helped to culture cells; JL helped to culture viruses; YX D had a co-ordination role in this work. All authors read and approved the final manuscript.
